# Apple procyanidins promote mitochondrial biogenesis and proteoglycan biosynthesis in chondrocytes

**DOI:** 10.1038/s41598-018-25348-1

**Published:** 2018-05-08

**Authors:** Isao Masuda, Masato Koike, Shohei Nakashima, Yu Mizutani, Yusuke Ozawa, Kenji Watanabe, Yoko Sawada, Hiroshi Sugiyama, Atsushi Sugimoto, Hidetoshi Nojiri, Koichi Sashihara, Koutaro Yokote, Takahiko Shimizu

**Affiliations:** 1 Department of Functional Materials Technology, Core Technology Laboratories, Asahi Group Holdings, Ltd., Ibaraki, Japan; 2 Department of Advanced Aging Medicine, Chiba University Graduate School of Medicine, Chiba, Japan; 3 Department of Clinical Cell Biology and Medicine, Chiba University Graduate School of Medicine, Chiba, Japan; 4 Products Development Department, Asahi Calpis Wellness Co., Ltd., Kanagawa, Japan; 5 Department of Orthopaedics, Juntendo University Graduate School of Medicine, Tokyo, Japan; 6 R & D Strategy Office, Asahi Group Holdings, Ltd., Tokyo, Japan; 7 Quality Assurance Department, Quality Assurance Headquarters, Asahi Group Foods, Ltd., Tokyo, Japan

## Abstract

Apples are well known to have various benefits for the human body. Procyanidins are a class of polyphenols found in apples that have demonstrated effects on the circulatory system and skeletal organs. Osteoarthritis (OA) is a locomotive syndrome that is histologically characterized by cartilage degeneration associated with the impairment of proteoglycan homeostasis in chondrocytes. However, no useful therapy for cartilage degeneration has been developed to date. In the present study, we detected beneficial effects of apple polyphenols or their procyanidins on cartilage homeostasis. An *in vitro* assay revealed that apple polyphenols increased the activities of mitochondrial dehydrogenases associated with an increased copy number of mitochondrial DNA as well as the gene expression of peroxisome proliferator-activated receptor gamma coactivator 1-α (PGC-1α), suggesting the promotion of PGC-1α-mediated mitochondrial biogenesis. Apple  procyanidins also enhanced proteoglycan biosynthesis with aggrecan upregulation in primary chondrocytes. Of note, oral treatment with apple procyanidins prevented articular cartilage degradation in OA model mice induced by mitochondrial dysfunction in chondrocytes. Our findings suggest that apple procyanidins are promising food components that inhibit OA progression by promoting mitochondrial biogenesis and proteoglycan homeostasis in chondrocytes.

## Introduction

Apple polyphenols are compounds of several polyphenols obtained from unripe apples. Growing evidence has shown that apple polyphenols have a radical scavenging activity^1,2^ and exhibit therapeutic efficacy, including anti-tumor^3^, anti-allergy^4^, anti-obesity^5^, anti-fatigue^6^, anti-dental cavity^7^, and life-extending effects^8^. The representative components of apple polyphenols are procyanidins, which are complex mixtures of the polymerized forms of (+)-catechin or (−)-epicatechin concatemers, leading to structural diversity (Supplementary Fig. S1). Procyanidins derived from a wide variety of fruits have also been reported to have antioxidative and anti-inflammatory activities^9–11^. The beneficial effects of apple polyphenols may be attributable to low-polymerized procyanidins, such as procyanidin B1, B2 and C1. Indeed, Shoji *et al*. detected procyanidin dimers or trimers in the serum of rats after the ingestion of a procyanidin fraction prepared from apple polyphenols, while highly polymerized procyanidins were not easily absorbed by the small intestine^12^.

Several groups have investigated the biological effects of procyanidins on the mitochondrial function. Procyanidin-rich polyphenols have been reported to show anti-tumor activities by inducing apoptosis through the mitochondrial pathway^3,13,14^. In addition, recent studies have indicated the effects of procyanidins on improving the mitochondrial quality using rat heart mitochondria^15^. Mizunoya *et al*. found that the oral intake of apple polyphenols upregulated the oxidative myosin heavy chain isoform MyHC IIx and shifted it to the oxidative fiber type, leading to the enhancement of the muscle endurance capacity in rats^16^. In our previous report using heart-specific mitochondrial dysfunction model mice, dietary apple polyphenols improved the survival and pathology of murine cardiomyopathy by decreasing the susceptibility to ventricular arrhythmias, suggesting that apple polyphenols might promote the mitochondrial function^2,17–19^.

Osteoarthritis (OA) is a common disease in the elderly due to an imbalance in cartilage matrix degradation and synthesis. Since the pathogenic mechanisms of OA are complicated and may be accelerated by the impairment of related tissues, such as cartilages, synovial tissues, bones and skeletal muscles with direct or indirect association, effective interventions have not yet been developed. Chondrocytes are cartilage-localized cells and responsible for producing, maintaining and degrading the extracellular matrix (ECM), which mainly consists of proteoglycan aggregates and collagen fibrils. Several groups have shown that the proteoglycan biosynthetic capacity or expression of anabolic genes was decreased in chondrocytes from OA patients^20–22^. Other groups have suggested a pathological relationship between mitochondrial superoxide dismutase 2 (SOD2) downregulation and cartilage degeneration in OA progression^23–25^. We also found that the specific loss of SOD2 in chondrocytes accelerated the mitochondrial redox imbalance and cartilage degeneration during aging using chondrocyte-*Sod2*^−/−^ mice^26^, suggesting a correlation between the mitochondrial function and proteoglycan homeostasis in chondrocytes.

In the present study, we evaluated the physiological role of apple polyphenols in the mitochondrial activity and proteoglycan synthesis in chondrocytes *in vitro*. Furthermore, the ability of apple procyanidins or procyanidin B2 isolated from apple polyphenols to protect articular cartilage was estimated using a mouse model of mitochondrial dysfunction-induced OA.

## Results

### Apple polyphenols promoted mitochondrial dehydrogenase activity and mitochondrial biogenesis in murine chondrocytes

**Figure 1:**
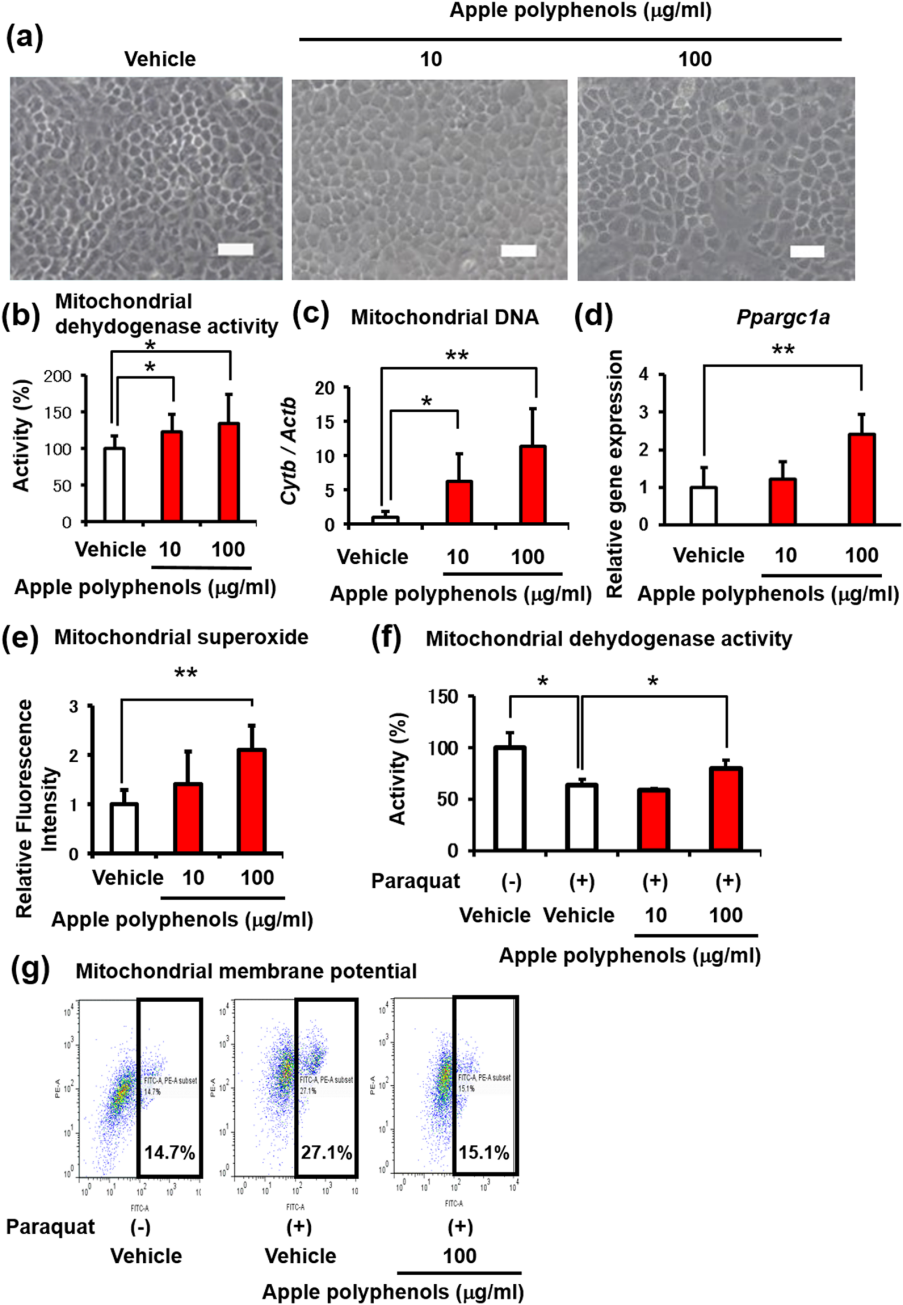
Apple polyphenols promote mitochondrial activity via mitochondrial biogenesis in primary chondrocytes. Apple polyphenols were added to primary chondrocytes or ATDC5 cells for 24 h. (**a**) Microscope images. Scale bars represent 100 µm. (**b**) Mitochondrial dehydrogenase activity (n = 9–10), (**c**) mitochondrial DNA level (n = 6), (**d**) gene expression of PGC-1α in ATDC5 cells (n = 6), (**e**) mitochondrial superoxide (n = 4–5). Primary chondrocytes were treated with apple polyphenols and paraquat. (**f**) Mitochondrial dehydrogenase activity (n = 3), (**g**) mitochondrial membrane potential (ΔΨ m). High: the region of cells with normal ΔΨ m, Low: the region of cells with mitochondrial depolarization. Values are the mean ± standard deviation (*P < 0.05, **P < 0.01, versus control, Student’s *t*-test).

In a previous report, procyandins were positively detected at 1.38–11.4 μg/ml in rat blood samples after the oral administration of 10.5–1000 mg/kg of procyanidins^12,27^. In this context, to investigate the cellular effects of apple polyphenols, which include abundant procyanidins, we added 10–100 μg/ml of apple polyphenols to murine proliferating chondrocytes. We observed non-toxic effects of apple polyphenols on primary chondrocytes (Fig. 1a), confirming our previous finding using PC-12 cells^28^. Next, we measured the cell viability and mitochondrial activity of apple polyphenols using an methyl thiazolyl tetrazolium (MTT) assay^29,30^. Apple polyphenols significantly promoted mitochondrial dehydrogenase activity in a dose-dependent manner in primary chondrocytes (Fig. 1b), although they did not alter the cell number (data not shown). To investigate the biological effect of apple polyphenols on the mitochondria of chondrocytes, the copy number of mitochondrial DNA was analyzed in the presence of apple polyphenols. Of note, apple polyphenols significantly increased the mitochondrial DNA and mitochondrial superoxide level, concomitant with promoting the gene expression of PGC-1α, which is the master regulator of mitochondrial biogenesis, indicating enhancement of mitochondrial biogenesis (Fig. 1c–e). Pharmacological experiments revealed that pretreatment of apple polyphenols attenuated decreases in the mitochondrial dehydrogenase activity induced by paraquat, a mitochondrial toxin, in primary chondrocytes (Fig. 1f). In addition, the proportion of cells with low mitochondrial membrane potentials was normalized in the presence of apple polyphenols, indicating improvement of mitochondrial depolarization impaired by paraquat (Fig. 1g). These data suggested that apple polyphenols promoted the mitochondrial activity as well as biogenesis in primary chondrocytes.

### Apple polyphenols modulated the mitochondrial function of Sod2^−/−^ chondrocytes with promoting mitochondrial biogenesis

**Figure 2:**
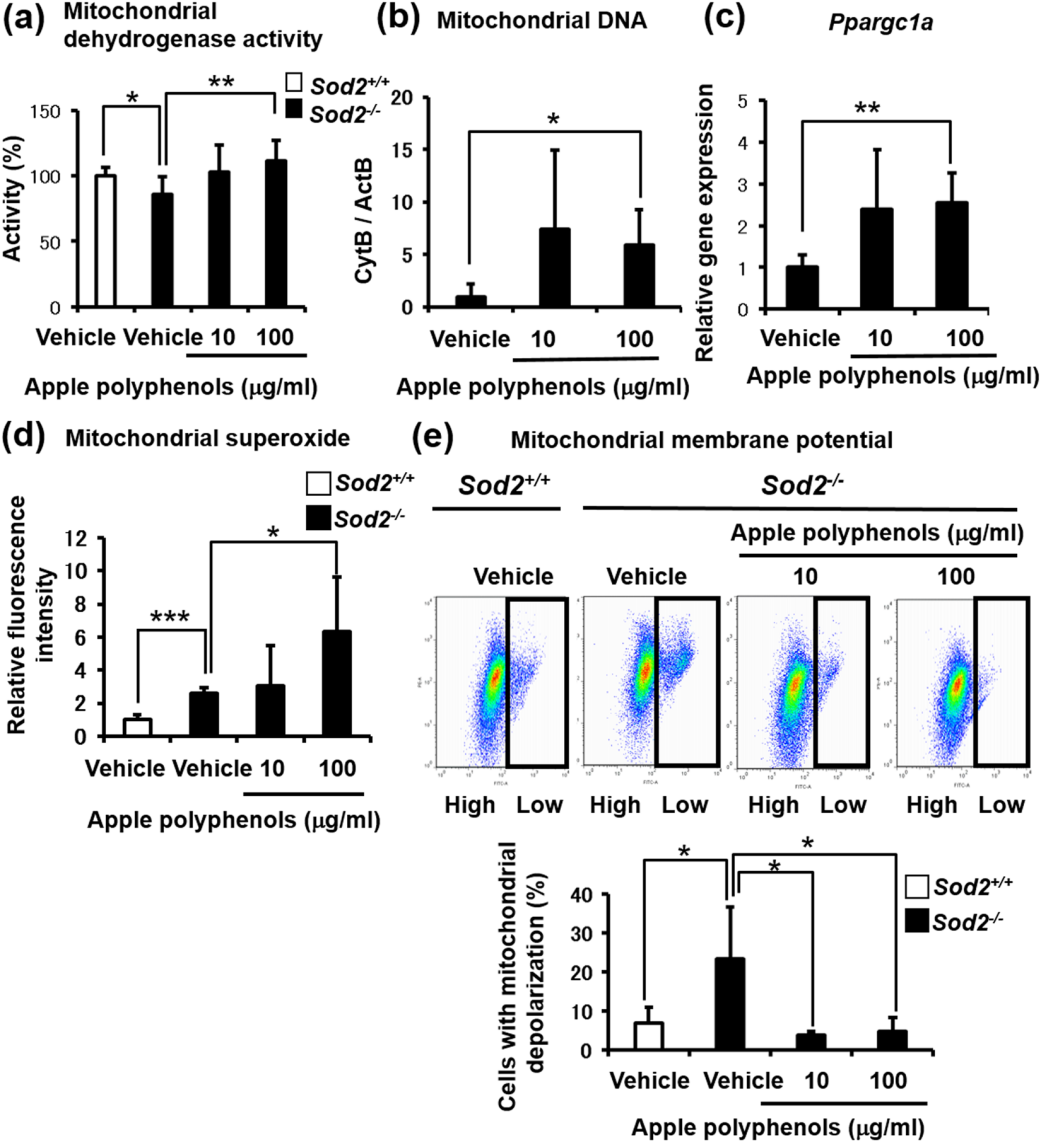
Apple polyphenols attenuate mitochondrial dysfunction through mitochondrial biogenesis in Sod2-deficient chondrocytes. Apple polyphenols were added to primary or *Sod2-*deficient murine chondrocytes for 24 h. (**a**) Mitochondrial dehydrogenase activity (n = 6–8), (**b**) mitochondrial DNA level (n = 3–5), (**c**) gene expression of PGC-1α (n = 4), (**d**) mitochondrial superoxide (n = 5–6), (**e**) mitochondrial membrane potential (ΔΨ m) (n = 3–5). Values are the mean ± standard deviation (*P < 0.05, **P < 0.01, ***P < 0.001 versus control, Student’s *t*-test).

To evaluate the protective effect of apple polyphenols on OA-related chondrocytes with mitochondrial dysfunction, we generated chondrocyte-*Sod2*^−/−^ mice and isolated *Sod2*^−/−^ chondrocytes from neonate mice for an *in vitro* assay. In our previous study, *Sod2*^−/−^ chondrocytes showed a disturbed mitochondrial function with mitochondrial redox imbalance^26^. Apple polyphenols did not change the morphology of *Sod2*^−/−^ chondrocytes (Supplementary Fig. S2). An MTT assay revealed that apple polyphenols promoted mitochondrial dehydrogenase activity in *Sod2*^−/−^ chondrocytes, indicating an increased cellular viability (Fig. 2a). In addition, apple polyphenols also increased the copy number of mitochondrial DNA, the gene expression of PGC-1α and mitochondrial superoxide in *Sod2*^−/−^ chondrocytes (Fig. 2b–d). Notably, apple polyphenols improved the mitochondrial depolarization impaired by *Sod2* loss (Fig. 2e). These findings demonstrated that apple polyphenols modulated the mitochondrial function and biogenesis associated with PGC-1α upregulation in OA-related chondrocytes.

### Apple procyanidins promoted proteoglycan synthesis in primary chondrocytes

**Figure 3:**
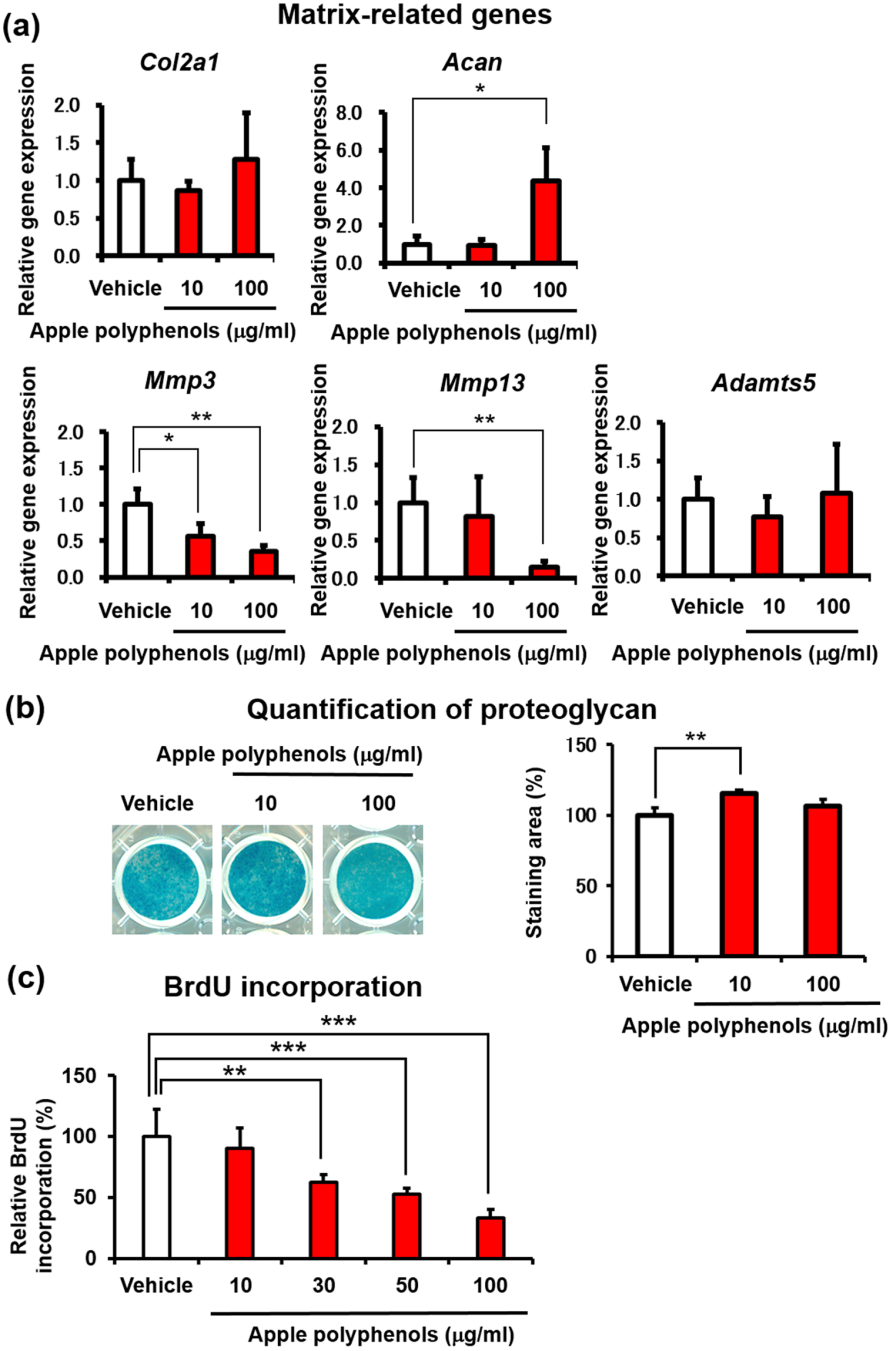
Apple polyphenols enhance proteoglycan synthesis in primary chondrocytes. Apple polyphenols were added to primary chondrocytes for 24 h for the assessment of their effects on the expression of matrix-related genes and BrdU incorporation, and for 14 days for the assessment of their effects on proteoglycan levels. (**a**) The expression profiles of matrix-related genes (n = 3), (**b**) proteoglycan levels (n = 3), (**c**) BrdU incorporation (n = 3). Values are the mean ± standard deviation (*P < 0.05, **P < 0.01, ***P < 0.001 versus control, Student’s *t*-test).

In order to clarify the biological effect of apple polyphenols on the ECM homeostasis in chondrocytes, we analyzed the expression of its related genes. Apple polyphenols significantly upregulated the anabolic gene *Acan* and downregulated the catabolic genes *Mmp3* and *Mmp13* (Fig. 3a). We further quantified the proteoglycan levels via Alcian blue staining at culture day 21 after treatment of apple polyphenols for 14 days. As expected, apple polyphenols significantly promoted proteoglycan synthesis in primary chondrocytes, possibly via *Acan* upregulation (Fig. 3a,b).

From their initial development to terminal differentiation, chondrocytes are known to undergo several steps of proliferation. To assess the effects of apple polyphenols on chondrocytic differentiation, we measured the incorporative activity of bromodeoxyuridine (BrdU). Apple polyphenols significantly decreased the BrdU incorporation in a dose-dependent manner in primary chondrocytes, indicating the promotive effects of apple polyphenols for proteoglycan biosynthesis in differentiated chondrocytes (Fig. 3c).

**Figure 4:**
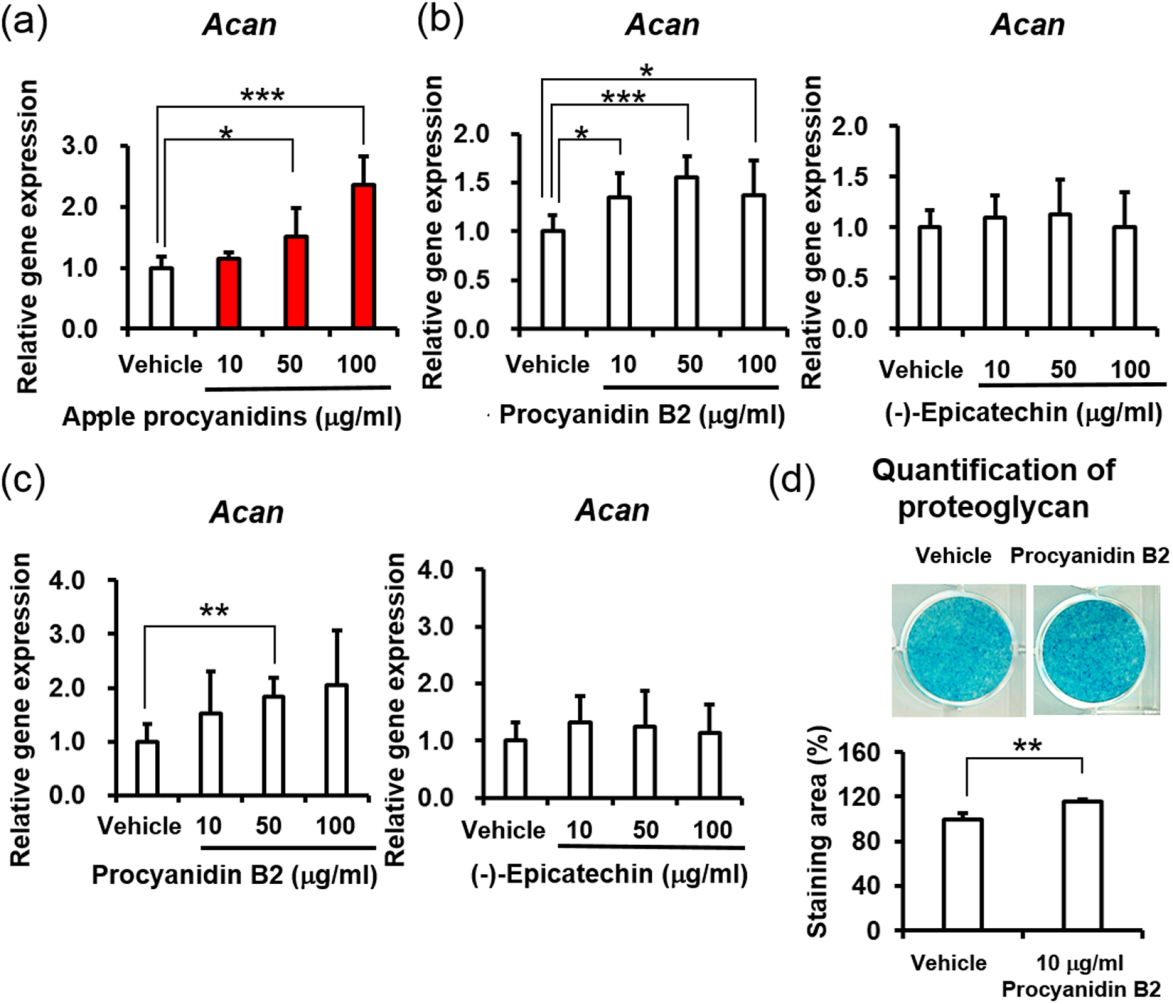
Apple procyanidins promote proteoglycan biosynthesis in murine chondrocytes. Apple procyanidins, procyanidin B2 and (−)-epicatechin were added to ATDC5 cells (**a**, **b**) or primary chondrocytes (**c**, **d**). (**a**–**c**) The gene expression of Aggrecan (n = 5–6). (**d**) Proteoglycan levels when treated with 10 μg/ml procyanidin B2 (n = 3). Values are the mean ± standard deviation (*P < 0.05, **P < 0.01, ***P < 0.001 versus control, Student’s *t*-test).

An HCl-butanol assay showed that procyanidins were major components of apple polyphenols (Table 1). Thus, we fractionated apple procyanidins and evaluated their activities in proteoglycan biosynthesis. Apple procyanidins also promoted the expression of *Acan in vitro* (Fig. 4a). Next, we validated the results to clarify the relationship between the structural characteristics of procyanidins and the potential for proteoglycan synthesis, since low-polymerized procyanidins have exhibited various beneficial effects^2,15,31,32^. In order to focus on their effects, we isolated procyanidin B2 and (−)-epicatechin monomer, which are abundant components and which are known to transfer into the bloodstream^12^, using phased fractionating methods^33,34^. Interestingly, procyanidin B2 enhanced the expression of *Acan* in ATDC5 cells and primary chondrocytes in a dose-dependent manner, while (−)-epicatechin monomer failed to promote the expression (Fig. 4b,c). Finally, we confirmed that long-term procyanidin B2 treatment was capable of regulating proteoglycan biosynthesis (Fig. 4d). These results indicated that the dimeric structure of procyanidin B2 in apple polyphenols played a pivotal role in the proteoglycan synthesis in chondrocytes. Based on these findings, we concluded that procyanidin B2 regulated proteoglycan homeostasis in murine chondrocytes.

**Table 1:** Components of polyphenols contained in apple polyphenols.

Compound	Content (%)	Method
Procyanidins	66.5	HCl-butanol assay
2-mer	4.7	Diol phase HPLC
Procyanidin B1	0.5	RP UPLC
Procyanidin B2	3.2	RP UPLC
3-mer	4.0	Diol phase HPLC
Procyanidin C1	1.0	RP UPLC
4-mer	1.9	Diol phase HPLC
5-mer	0.4	Diol phase HPLC
(+)−Catechin	0.5	RP UPLC
(−)−Epicatechin	3.4	RP UPLC
Chlorogenic acid	6.5	RP UPLC
p-Coumaroyl quinic acid	2.8	RP UPLC
Phloridzin	1.5	RP UPLC
Phloretin xylosylglucoside	3.6	RP UPLC

The total procyanidins and procyanidin oligomers were measured by HCl-butanol assay and diol phase HPLC, respectively. The quantity of each component was analyzed by reversed phase (RP) UPLC individually.

### Apple procyanidins significantly ameliorated the cartilage damage in the knee joints of chondrocyte-Sod2^−/−^ mice under mechanical overloading

To evaluate whether or not apple procyanidins protect cartilage degeneration induced by mitochondrial dysfunction of chondrocytes, destabilization of the medial meniscus (DMM) surgery was performed on the left knees of chondrocyte*-Sod2*^−/−^ and their control littermates. After the surgery, we orally administered 500 mg/kg of apple procyanidins and 100 mg/kg of isolated procyanidin B2, and the cartilage damage was histologically evaluated in safranin O/fast green-stained sections of knee joints by the modified OARSI scoring system (Supplementary Table 2). To set the dose of procyanidins, we referred to previous studies about the bioavailability of procyanidins, in which procyanidins that were orally administered at a concentration of 10.5–1000 mg/kg were transferred to the bloodstream at 1.38–11.4 μg/ml^12,27^.

**Figure 5:**
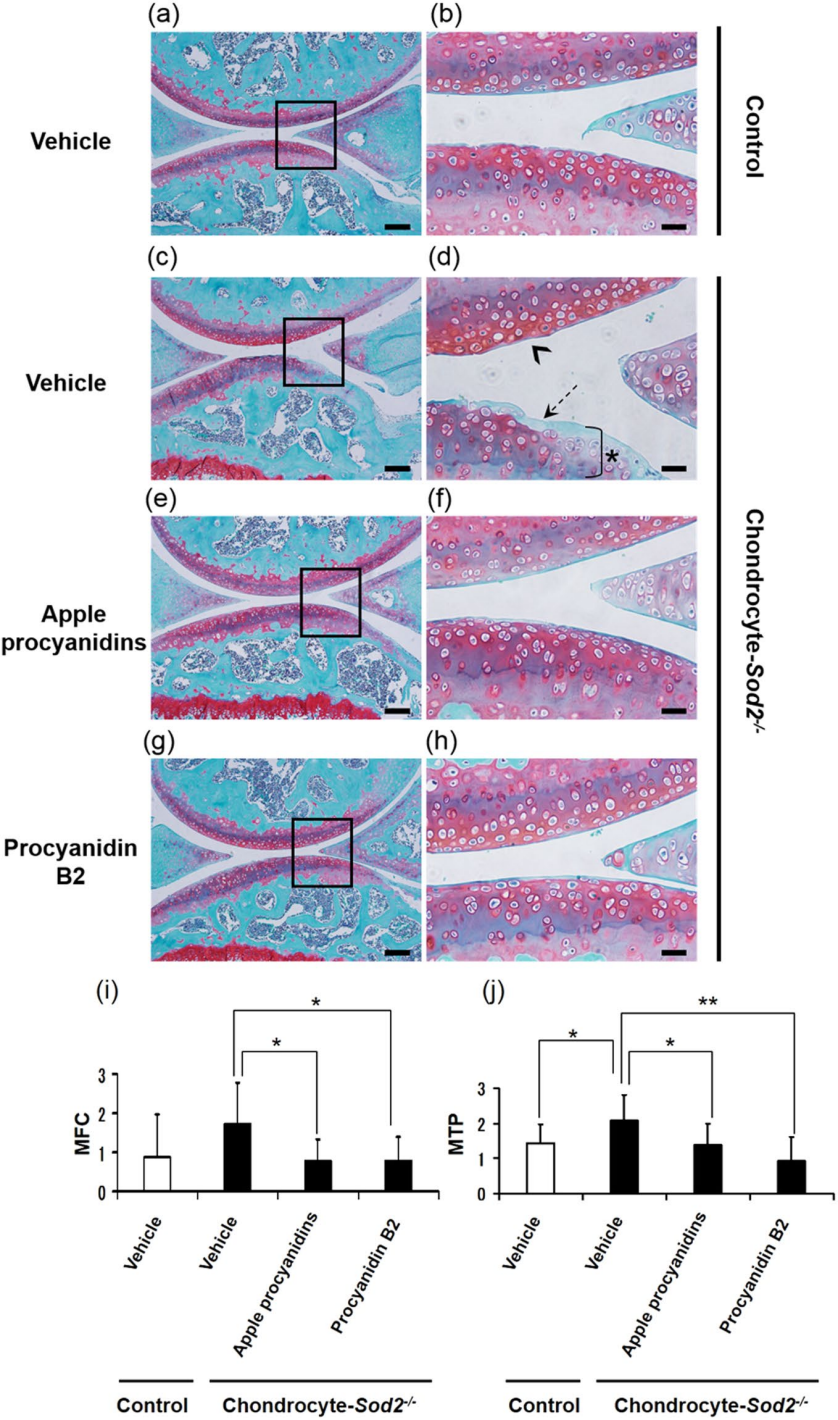
Apple procyanidins and procyanidin B2 suppress cartilage damages in knee joints of chondrocyte-*Sod2*^−/−^ mice under mechanical overloading. The left knee joints of mice were treated with DMM surgery at 8 weeks of age and administered 500 mg/kg apple procyanidins or 100 mg/kg procyanidin B2 for 8 weeks. (**a**–**h**) Cartilage degeneration in safranin O/fast green-stained sections of the medial region of knee joints from control and chondrocyte-*Sod2*^−/−^ mice. Scale bars represent 100 µm and 1 mm for the left and right panels, respectively. The arrowhead, dotted arrow, and asterisk represent cartilage fibrillation, cartilage degeneration/erosion, and loss of safranin O intensity, respectively. (i and j) Quantification of cartilage degeneration in the medial region; the left graph indicates the quantification of cartilage degeneration in the medial femoral condyle (MFC), and the right graph indicates the quantification of cartilage degeneration in the medial tibial plateau (MTP). Values are the mean ± standard deviation (n = 10–12, *P < 0.05, **P < 0.01 versus control, Student’s *t*-test).

We confirmed that chondrocyte*-Sod2*^−/−^ joints exhibited cartilage degeneration or erosion, especially in the medial side of tibial plateau, compared with control joints following DMM surgery (Fig. 5b–d). Interestingly, we found that the oral treatment of apple procyanidins and procyanidin B2 significantly decreased the cartilage damage scores of chondrocyte*-Sod2*^−/−^ joints at the medial side of the femoral condyle (MFC) and tibial plateau (MTP) (Fig. 5). At the femoral side, apple procyanidins and procyanidin B2 attenuated the fibrillation and roughness of the cartilage surfaces (arrowhead in Fig. 5d,f and h). Apple procyanidins and procyanidin B2 also ameliorated the cartilage degeneration or erosion with the loss of safranin O intensity to the tidemark level at the tibial side (dotted arrow and asterisk in Fig. 5,f–h), resembling the morphology of the control cartilage (Fig. 5b). In addition, apple procyanidins and procyanidin B2 tended to normalize the surfacial safranin O loss in *sham* cartilage of chondrocyte*-Sod2*^−/−^ mice, although no significant differences were observed (Supplementary Fig. S3, solid arrow).

Apple procyanidins and procyanidin B2 did not modify the body weight or spontaneous locomotive activity in these mice, suggesting that treatment with apple procyanidins showed fairly few adverse effects on the cartilage maintenance *in vivo* (Supplementary Fig. S4). These results showed that apple procyanidins ameliorated the cartilage damage to murine knee joints exacerbated by *Sod2* depletion in chondrocytes under mechanical overloading.

## Discussion

### Apple polyphenols modulated the mitochondrial activity and biogenesis in murine chondrocytes

In this study, an apple polyphenol concentration of 10–100 μg/ml was adopted based on previous reports about the physiology^3,8,31^ or availability^12,27^ of procyanidins. We demonstrated that apple polyphenols increased mitochondrial dehydrogenase activities and the copy number of mitochondrial DNA in murine chondrocytes concomitant with *Pgc-1α* upregulation (Figs 1 and 2). Accumulating evidence strongly suggests that the activation of PGC-1α is associated with mitochondrial biogenesis^35,36^. Yun *et al*. showed that the mitochondrial biogenesis and PGC-1α expression were impaired in OA chondrocytes, but was pharmacologically reversible by increasing the PGC-1α expression^37^. We previously reported that apple polyphenols and their procyanidins extended the lifespan of *Caenorhabditis elegans* in a SIR-2 (an orthologue of SIRT1)-dependent manner^8^. Several groups have also reported the induction of PGC-1α by some food ingredients through co-activating SIRT1, AMPKα, NRF-1 and TFAM^38–41^. Furthermore, Choi *et al*. showed the selective inhibitory activities of procyanidin B3 for histone acetyltransferase (p300)^42^. We therefore proposed that apple polyphenols directly or indirectly promoted PGC-1α expression, leading to mitochondrial biogenesis in chondrocytes.

Apple polyphenols improved mitochondrial depolarization concomitant with mitochondrial biogenesis despite *Sod2* deficiency (Fig. 2e). These data suggested that the promoting effects of apple polyphenols on mitochondrial biogenesis might improve the mitochondrial quality under conditions of attenuated mitochondrial depolarization in chondrocytes. Indeed, Hasegawa *et al*. demonstrated that the promotion of mitochondrial biogenesis exerted neuroprotective effects against mitochondrial insults^43^. Further analyses are required to clarify how to improve the mitochondrial membrane potentials by treatment of apple polyphenols.

We showed that apple polyphenols increased the mitochondrial superoxide level in murine chondrocytes while exerting a variety of protective effects (Figs 1–4). Increasing evidence has suggested mitohormetic effects^44–46^, wherein reactive oxygen species do not only cause oxidative stress but also function in the promotion of mitochondrial biogenesis^47–49^, which is consistent with our data in Figs 1 and 2. Although the potential mechanisms underlying the effects of procyanidins on the superoxide levels in chondrocytes remain to be clarified, apple procyanidins might activate the mitohormetic pathway.

### A procyanidin dimer characteristically promoted proteoglycan biosynthesis in chondrocytes

Other groups have reported a correlation between mitochondrial activity and proteoglycan homeostasis^50,51^. Using cartilage explants from normal human donors, Pastor *et al*. indicated that mitochondrial respiratory dysfunction inhibited proteoglycan homeostasis, suggesting that mitochondria in chondrocytes play important roles in maintaining healthy cartilage^51^. We discovered that apple polyphenols promoted proteoglycan synthesis associated with accelerating the mitochondrial function and modulating the expression of ECM-related genes in primary chondrocytes (Figs 1–3, Supplementary Fig. S5). Interestingly, we also found that the procyanidin fraction of apple polyphenols and isolated procyanidin B2 promoted the expression of *Acan*, while (−)-epicatechin did not influence the expression level at all (Fig. 4, Supplementary Fig. S6). Regarding the specific effects of procyanidin oligomers, Nishizuka *et al*. demonstrated that apple procyanidin oligomers more strongly associated with LOX-1 protein, which oxidized LDL receptor, than did (−)-epicatechin in LOX-1-CHO cells^32^. Furthermore, Takahashi *et al*. suggested that procyanidin dimers and a trimer promoted the growth of murine keratinocytes more intensively than the (−)-epicatechin^31^. These findings support the notion that a dimeric structure of procyanidin is required for the exertion of its biological effects, such as proteoglycan synthesis.

### Apple procyanidins decreased the proliferation and accelerated the differentiation of primary chondrocytes

We observed the upregulating effects of apple polyphenols on the expression of the representative anabolic gene *Acan* and the downregulating effects on BrdU incorporation in primary chondrocytes (Fig. 3a,c). Other groups have shown that *Acan* expression transiently increased at the beginning of hypertrophy in an experiment using bovine growth plates^52^. In general, chondrocytes cease proliferation prior to hypertrophy, which is the terminal step of differentiation. Transition from the proliferative phase to hypertrophic differentiation is a crucial step for promoting proteoglycan synthesis in chondrocytes. Indeed, Hirata *et al*. reported that the transcription factor CCAT/enhancer binding protein β (C/EBPβ) inhibited proliferation, as measured by a BrdU assay, and promoted hypertrophic differentiation in primary chondrocytes^53^. Furthermore, using chondrocytes from the cartilage of adult pigs, Grandolfo *et al*. reported an increase in the number of mitochondria per cell concomitant with their hypertrophic differentiation^54^, as we showed in Figs 1 and 2. These findings, taken together with the beneficial effects of proteoglycan homeostasis, suggest that apple procyanidins may promote proteoglycan synthesis by inducing hypertrophic differentiation in chondrocytes.

### Apple procyanidins protected against articular cartilage degeneration in OA model mice

Approaches to treating the articular cartilage via the oral intake of plant materials or plants’ polyphenols have been reported in previous studies. In 2014, Leong *et al*. demonstrated the protective effects of epigallocatechin 3-gallate, a major polyphenol in green tea, for murine articular cartilage degeneration with an imbalance in proteoglycan homeostasis induced by DMM surgery^55^. A clinical trial for healthy people with a moderate loss of the joint function showed that dietary apple procyanidins in the peel powder improved their function and reduced associated pain^56^. Regarding the application of procyanidins for OA, Aini *et al*. reported the protective effects of the grape seed procyanidin B3 for cartilage degeneration in knee joints of OA model mice, suggesting the protective effects of procyanidin B3 for H_2_O_2_-induced chondrocytic apoptosis or iNOS expression in synovial tissues^57^. In the present study, we demonstrated that the treatment of apple procyanidins or procyanidin B2 significantly prevented cartilage fibrillation, degeneration and erosion as well as the loss of safranin O staining in knee joints of chondrocyte-*Sod2*^−/−^ mice under conditions of instability (Fig. 5). We confirmed the downregulation of some OA-related genes in the infrapatellar fat pad, which include synovial tissues, of the operated side of chondrocyte-*Sod2*^−/−^ mice at two weeks after DMM surgery (Supplementary Fig. S7). The reduction in the effects of procyanidin B2 on the *iNOS* expression in the infrapatellar fat pad was consistent with the findings of previous reports using procyanidin B3^57^. In the non-operated knee joints, we observed that apple procyanidins or procyanidin B2 moderated surface changes in the cartilages (Supplementary Fig. S2). Given these findings, along with those in *in vitro* studies, we proposed that the oral administration of apple procyanidins protected against articular cartilage degeneration and prevented the development of knee OA in chondrocyte-*Sod2*^−/−^ mice under mechanical overloading due to the modulation of mitochondrial biogenesis and proteoglycan biosynthesis in chondrocytes.

However, there are some limitations with regard to applying the results of this study to clinical trials. Although we confirmed that there were no significant gender differences in articular cartilage degeneration in our mutant models, we also need to validate the effects in females. Moreover, the doses of apple procyanidins and procyanidin B2 that were administered (500 mg/kg and 100 mg/kg body weight, respectively), are high for humans and the applicability of our results to clinical trials might be limited. As the next stage, further analyses should be performed to clarify the protective effects against cartilage degeneration and the dosages of apple procyanidins that are appropriate for clinical trials.

In this study, we showed that apple polyphenols and their procyanidins exerted beneficial effects on chondrocytes and murine articular cartilage concomitant with the enhancement of mitochondrial biogenesis and promotion of proteoglycan biosynthesis. The mitochondrial-promoting pathway of procyanidins proposed herein may provide new insight into the potential mechanisms underlying the effects of procyanidins. Our findings strongly suggest that apple polyphenols are promising food components for maintaining healthy cartilage.

## Methods

### Preparation of apple polyphenols

Apple polyphenols were prepared from unripe apples (*Malus pumila* x *domestica Borkh. cv. Fuji*) according to the method of Shoji *et al*. with slight modification^33^. In brief, unripe apple juice was subjected to solid phase extraction with SEPABEADS SP-70 (Mitsubishi Chemical Corporation, Tokyo, Japan). The eluate was condensed and spray-dried to obtain powdered apple polyphenols.

### Isolation of procyanidin species from apple polyphenols

Procyanidins are polymeric compounds of catechin or epicatechin and thus exist as a complex mixture of many isomers that are difficult to isolate by simple reversed phase high-performance liquid chromatography (RP-HPLC). Apple polyphenol powder was dissolved in deionized water and adjusted to pH 7.0 and then loaded onto a column filled with Diaion HP-20 (Mitsubishi Chemical Corporation). Adsorbed apple procyanidins were rinsed with distilled H_2_O, followed by elution with EtOH-H_2_O (21:79, w/w). The corresponding eluate was concentrated and spray-dried to obtain the powdered apple procyanidins. Procyanidins with a low degree of polymerization were extracted from the apple procyanidins with methyl acetate, and the spray-dried extract was fractionated according to the degree of polymerization by semi-preparative HPLC with a diol phase column, as reported by Nakashima *et al*.^34^. Procyanidin monomer and dimer fractions were further purified by the method of Shoji *et al*. to obtain (–)-epicatechin and procyanidin B2^33^. The purity of these compounds was confirmed to be over 95% based on the ratio of the RP-HPLC peak area. Purified (–)-epicatechin and procyanidin B2 were freeze-dried and kept at −30 °C. Apple polyphenols or their isolated components were resolved in PBS as a stock solution for the *in vitro* study; they were then diluted to the target concentrations with culture medium.

### Generation of chondrocyte-Sod2^−/−^ mice

Chondrocyte-*Sod2*^−/−^ mice were generated by crossbreeding *Sod2*^*fl/fl*^ mice on a C57BL/6NCrSlc with *Col2a1* promoter-*Cre* transgenic mice on a C57BL6/J as previously reported^26,58–60^. Obtained wild-type (*Sod2*^*fl/fl*^) or chondrocyte-*Sod2*^−/−^ (*Col2a1-Cre;Sod2*^*fl/fl*^) mice were supplied for cell cultures or histological evaluations.

### Cell culture of primary articular chondrocytes with apple polyphenols or procyanidins

Primary articular chondrocytes were prepared from 6-day-old pups of wild-type or chondrocyte-*Sod2*^−/−^ mice as previously described with some modifications^61,62^. Primary chondrocytes were seeded at a density of 8,000 cells/cm^2^ in plastic dishes and cultured with medium (consisting of α-MEM supplemented with 10% fetal bovine serum [FBS, Thermo Fisher Scientific, Waltham, MA, USA], 100 units/mL penicillin, and 0.1 mg/mL streptomycin) at 37 °C in a 20% O_2_ and 5% CO_2_ incubator. When cells reached confluence, the medium was refreshed with apple reagents. Cells incubated with apple polyphenols, procyanidins or PBS as a vehicle for 24 h were supplied for the assessment of the cellular morphology, mitochondrial dehydrogenase activity, copy number of mitochondrial DNA, gene expressions, mitochondrial superoxide, mitochondrial membrane potential. At culture day 21, cultured cells were used for the quantification of proteoglycan synthesis.

### Cellular morphology of primary articular chondrocytes

Primary articular chondrocytes that reached confluence were observed using an inverted microscope (LEICA DMIRB) (Leica, Wetzlar, Germany) under 10× magnification.

### Measurement of mitochondrial dehydrogenase activity or cellular proliferation

Mitochondrial dehydrogenase activity was measured by an MTT assay (Dojindo Laboratories, Kumamoto, Japan). Cell proliferation was measured using a BrdU enzyme-linked immunosorbent assay (ELISA) kit (Roche Diagnostics K.K., Tokyo, Japan) according to the manufacturer’s instructions.

### Quantification of mitochondrial DNA by genomic PCR

Genomic DNA was extracted from primary chondrocytes. Cultured primary chondrocytes were incubated with 500 µg/mL proteinase K overnight at 37 °C. After the reaction, chondrocytes were suspended with an equal amount of TRIzol (Life Technologies Corporation, Carlsbad, CA, USA), then incubated on ice for 5 min and centrifuged for 5 min at 12,000 *g* at 4 °C. The upper phase was placed into a new microtube and suspended with an equal amount of chloroform. After incubation on ice for 5 min, the solution was centrifuged for 5 min at 12,000 *g* at 4 °C. The obtained upper phase solution was agitated with an equal amount of sodium acetate/isopropanol (1:40, v/v) and incubated for 30 min at −80 °C and then centrifuged for 10 min at 12,000 *g* at 4 °C. After removing the supernatant, the pellet was agitated with 75% ethanol and centrifuged for 5 min at 7,500 *g* at 4 °C. The supernatant was removed, and the pellet was dried for 10 min at room temperature and then suspended with TE buffer to make a genomic DNA solution. Genomic DNA was used as a template for the genomic PCR analyses. Mitochondrial DNA was quantified using the MJ Mini thermal cycler (Bio-Rad Laboratories, Hercules, CA, USA) with SYBR Green Supermix (Bio-Rad Laboratories) according to the manufacturer’s instructions.

### Culture of ATDC5 cells with apple polyphenols or procyanidins

The mouse chondrogenic ATDC5 cell line was obtained from DS Pharma Biomedical (Osaka, Japan). Cells were cultured in the maintenance medium consisting of DMEM/F12 (1:1) medium containing 5% FBS, 10 µg/ml human transferrin, 3 × 10^−8^ M sodium selenite (Sigma-Aldrich, St. Louis, MO, USA) and 1% antibiotics (Thermo Fisher Scientific) at 37 °C in a 20% O_2_ and 5% CO_2_ incubator. ATDC5 cells were seeded in 12-well plates with 4 × 10^4^ cells per well. Chondrogenic differentiation was performed as previously described^57,63^. When cells reached confluence, the medium was replaced with maintenance medium supplemented with 10 µg/ml insulin (Sigma-Aldrich) and cultured for 4 days. Differentiated ATDC5 cells were treated with apple polyphenols, procyanidins or PBS for 24 h.

### An analysis of the gene expression by quantitative real-time PCR

Total RNA was extracted from cells or tissues with TRIzol according to the manufacturer’s instructions. Complementary DNA (cDNA) was synthesized from 1 μg of total RNA using the ReverTra Ace qPCR RT Kit (TOYOBO, Osaka, Japan). Total cDNA (100 ng) was used as a template for the real-time RT-PCR analyses. cDNA was quantified using the MJ Mini thermal cycler or Applied Biosystems 7500 Fast Real-Time PCR System (Applied BioSystems, Foster City, CA, USA) with SYBR Green as described above.

### Superoxide generation in primary articular chondrocytes

Cultured chondrocytes were stained with MitoSox (Life Technologies Corporation) for detection of mitochondrial superoxide as previously described with some modifications^26,64^. Superoxide generation was measured by a BD FACS Canto II flow cytometer (BD Biosciences, San Jose, CA, USA).

### Paraquat treatment

Methyl viologen dichloride hydrate (paraquat; Sigma-Aldrich) was dissolved in PBS to create a stock solution of 10 mM and used at a final concentration. On measuring the mitochondrial dehydrogenase activity, primary chondrocytes at culture day 5 were pretreated with apple polyphenols and then incubated with 500 µM paraquat for 24 h. For the assessment of the mitochondrial membrane potential, 5-day-cultured chondrocytes were incubated with apple polyphenols and 1 mM paraquat for 24 h.

### Measurement of the mitochondrial membrane potential

Primary articular chondrocytes were stained with JC-1 dye (Life Technologies Corporation) as previously described with some modifications^64^. The mitochondrial membrane potential was measured using a BD FACS Canto II flow cytometer (BD Biosciences).

### Quantification of Alcian blue staining

The proteoglycan production in chondrocytes was evaluated using Alcian blue (Muto Pure Chemicals, Tokyo, Japan) staining as previously described^62^. Stained chondrocytes were captured by a flathead scanner, and then images were quantified using the QWin image analysis software program (Leica).

### Surgical induction of OA and oral treatment of apple reagents

All experimental procedures were performed in accordance with specified guidelines for the care and use of laboratory animals and approved by the Animal Care and Use Committee of Chiba University. Wild-type or chondrocyte-*Sod2*^−/−^ male mice (8 weeks old) were divided into three groups: apple procyanidins, procyanidin B2 and vehicle control groups. The surgery-induced OA model was produced by resecting the medial meniscotibial ligament (MMTL) of the left knee joint as previously described^65^. The right knee underwent a *sham* operation. Five days after DMM surgery, apple procyanidins (500 mg/kg body weight), procyanidin B2 (100 mg/kg body weight) or the vehicle control was administered orally once a day. After 2 or 8 weeks, mice were sacrificed, and the infrapatellar fat pads or entire knee joints were obtained for the quantification of the synovial gene expression and evaluation of the histology of the cartilage, respectively.

### A histological evaluation of the knee joints in DMM model mice

The knee joints obtained were fixed in 4% paraformaldehyde plus 0.1 M PBS solution for 48 h at 4 °C, decalcified for 2 weeks with 20% EDTA∙2Na (Dojindo Laboratories) at 4 °C on a shaker, and embedded in paraffin wax. Paraffin sections were stained with safranin O and fast green. The histological OA grade was evaluated using the modified OARSI histopathology grading system^66^. The MFC and the MTP of each knee joint were scored individually, and three sagittal sections were averaged. OA grading was assessed by a single observer who was blinded to the study.

### Locomotive activity

Locomotive activity was monitored using implanted transmitting devices as previously described^67^. Scores were obtained as counts per hour, and the 24-h profile of the daily activity was obtained by averaging four days of continuous data.

### Statistical analyses

Data are expressed as the mean ± standard deviation. Statistical analyses were performed with Student’s *t*-test or Tukey’s test. *p* values < 0.05 were considered significant.

## Electronic supplementary material

Supplementary figures
