# Supplementary material for: Apple procyanidins promote mitochondrial biogenesis and proteoglycan biosynthesis in chondrocytes
Source: Sci Rep. 2018 May 8;8:7229. doi: 10.1038/s41598-018-25348-1 (PMC5940809; doi:10.1038/s41598-018-25348-1)
Supplement: Supplementary file 1 — Supplementary figures [file 41598_2018_25348_MOESM1_ESM.pdf]

Supplementary Online Material for

Apple procyanidins promote mitochondrial biogenesis and proteoglycan biosynthesis in chondrocytes

Isao Masuda, Masato Koike, Shohei Nakashima, Yu Mizutani,
Yusuke Ozawa, Kenji Watanabe, Yoko Sawada, Hiroshi Sugiyama,
Atsushi Sugimoto, Hidetoshi Nojiri, Koichi Sashihara,
Koutaro Yokote, Takahiko Shimizu

Correspondence should be addressed to Takahiko Shimizu (shimizu@chiba-u.jp)

Supplementary Table S1. Primers for real-time or genomic PCR

	Forward 5'-3'	Reverse 3'-5'
**For genomic PCR**		
Actb	AGCCATGTACGTAGCCATCCA	ACCAGAGGCATACAGGGACA
Cytb	GCCACCTTGACCCGATTCT	TTGCTAGGGCCGCGATAAT
**For real-time PCR (primary chondrocytes)**		
Gapdh	ATGTGTCCGTCGTGGATCTGA	TGCCTGCTTCACCACCTTCT
Ppargc1a	GAAAGGGCCAAACAGAGAGA	GTAAATCACACGGCGCTCTT
Col2a1	GGCAACAGCAGGTTCACATA	ATGGGTGCGATGTCAATAAT
Acan	CAGAGTTAGTGGAGGGTGTGA	AGACCCTGGGAAGTTTGT
Mmp3	TGTGTGCTCATCCTACCCATTGC	CCCTGTCATCTCCAACCCGAGGA
Mmp13	AGGCCTTCAGAAAAGCCTTC	TCCTTGGAGTGATCCAGACC
Adamts5	CCTGGCGGTGGTGAAGGTGG	TGCCCACATAAATCCTCTCGGGTGA
Sod2	CTGGACAAACCTGAGCCCTAAG	AAGACCCAAAGTCACGCTTGA
iNOS	ACATCGACCCGTCCACAGTAT	CAGAGGGGTAGGCTTGTCTC
Il1b	ATGGCAACTGTTCCTGAACTCAACT	CAGGACAGGTATAGATTCTTTCCTTT
Ptgs2	CTGCTGCCCGACACCTTCAACA	CATTTCTTCCCCCAGCAACCCGG
**For real-time PCR (ATDC cells)**		
Gapdh	ATGGCCTTCCGTGTTCCTAC	TGCCTGCTTCACCACCTTC
Ppargc1a	CGGAAATCATATCCAACCAG	TGAGGACCGCTAGCAAGTTTG

Supplementary Table S2. The semi-quantitative scoring system of articular cartilage damage

Score	Arthritis damage (*most significant damage)
0	Normal
0.5	Loss of safranin O without structural changes
1	Small fibrillation without loss of cartilage, not including loss of safranin O to tidemark level
2	Vertical clefts/erosion to the cartilage layer expending to <10% from the articular surface, including loss of safranin O to tidemark level
3	Vertical clefts/erosion to the cartilage layer expending to 10-25% from the articular surface
4	Vertical clefts/erosion to the cartilage layer expending to 25-50% from the articular surface
5	Vertical clefts/erosion to the cartilage layer expending to 50-75% from the articular surface
6	Vertical clefts/erosion to the cartilage layer expending to >75% from the articular surface

The OARSI scoring system was modified.

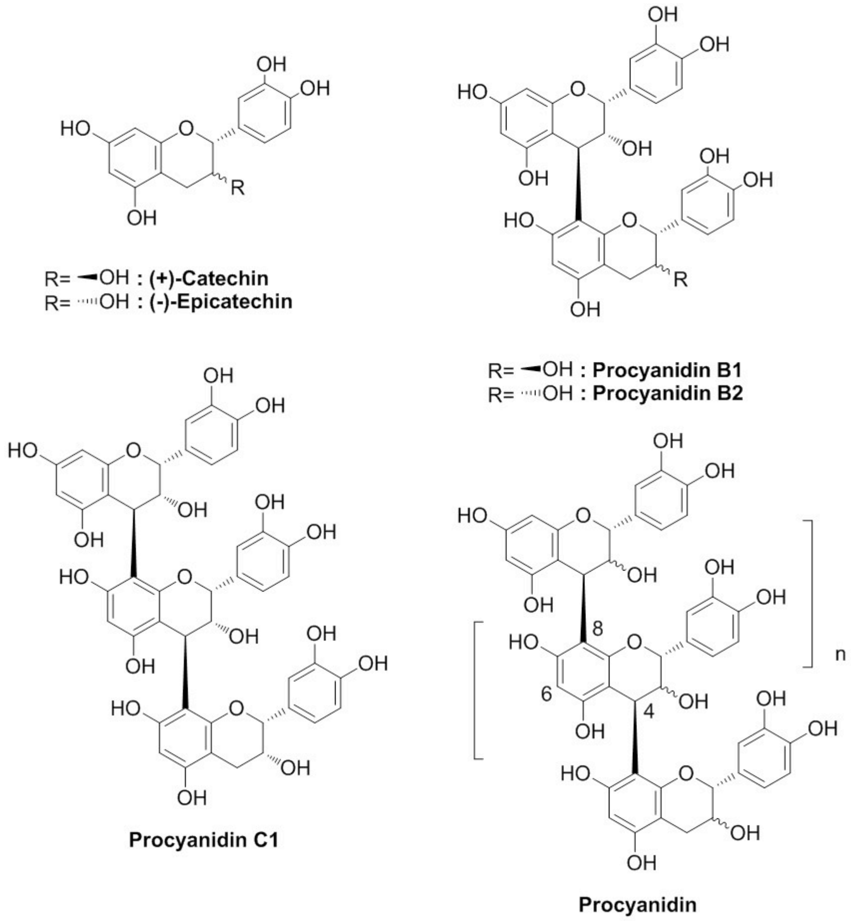

Supplementary Figure S1. Structures of major apple procyanidins and basic procyanidins.

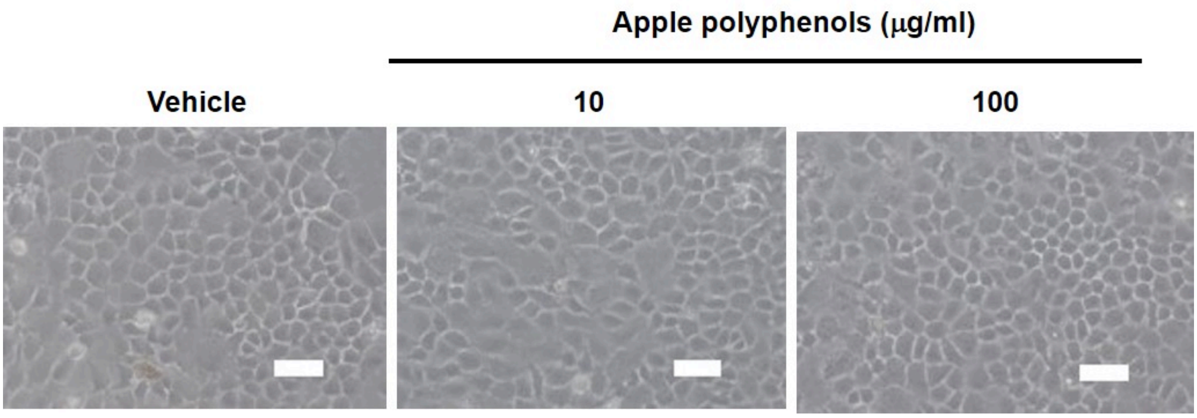

Supplementary Figure S2. Apple polyphenols do not change the morphology of *Sod2*$^{-/-}$ chondrocytes. Apple polyphenols were added to *Sod2* $^{-/-}$ chondrocytes for 24 h. Scale bars represent 100 μm.

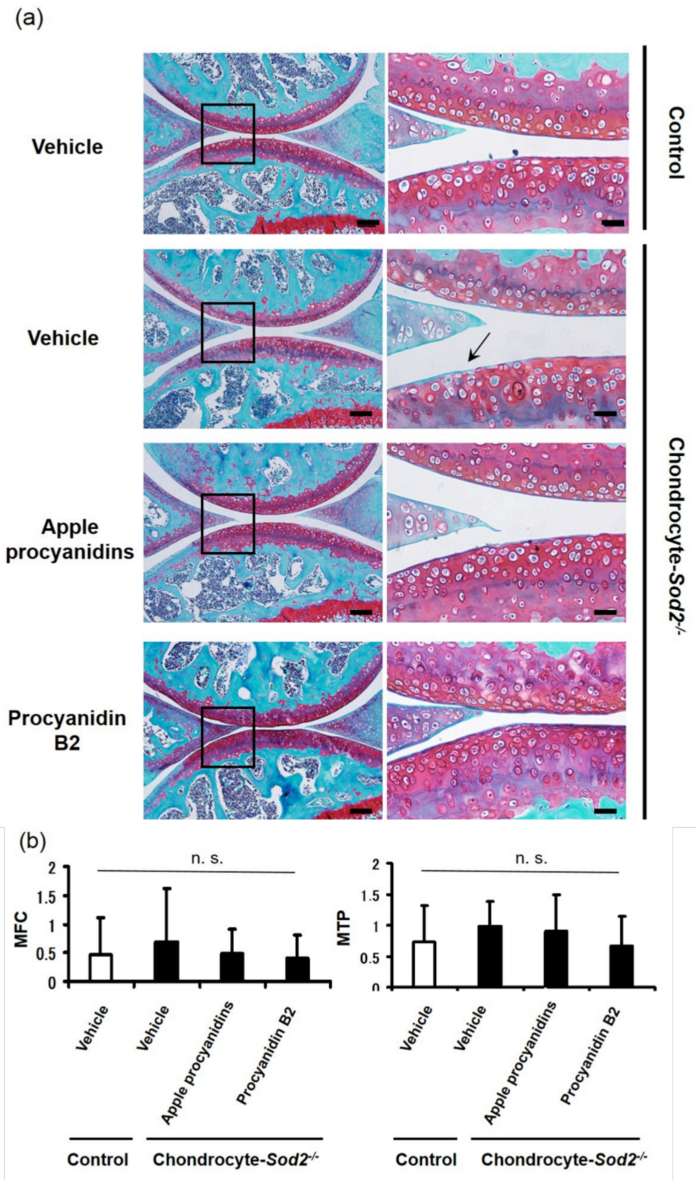

Supplementary Figure S3. Apple procyanidins or procyanidin B2 do not affect the cartilage damage to the *sham*-operated knee joints of chondrocyte-*Sod2*$^{-/-}$ mice under mechanical overloading. Right knee joints of mice were treated with *sham* operations at 8 weeks of age and administered 500 mg/kg apple procyanidins or 100 mg/kg procyanidin B2 for 8 weeks. (a) Cartilage degeneration in safranin O/fast green-stained sections of the medial region of knee joints from control and chondrocyte-*Sod2*$^{-/-}$ mice. Scale bars represent 100 µm and 1 mm for the left and right panels, respectively. The solid arrow indicates the loss of Safranin O intensity. (b) Quantification of cartilage degeneration in the medial region; the left graph indicates the quantification of cartilage degeneration in the medial femoral condyle (MFC), and the right graph indicates the quantification of cartilage degeneration in the medial tibial plateau (MTP). Values are the mean ± standard deviation (n = 10-12, Student's *t*-test)

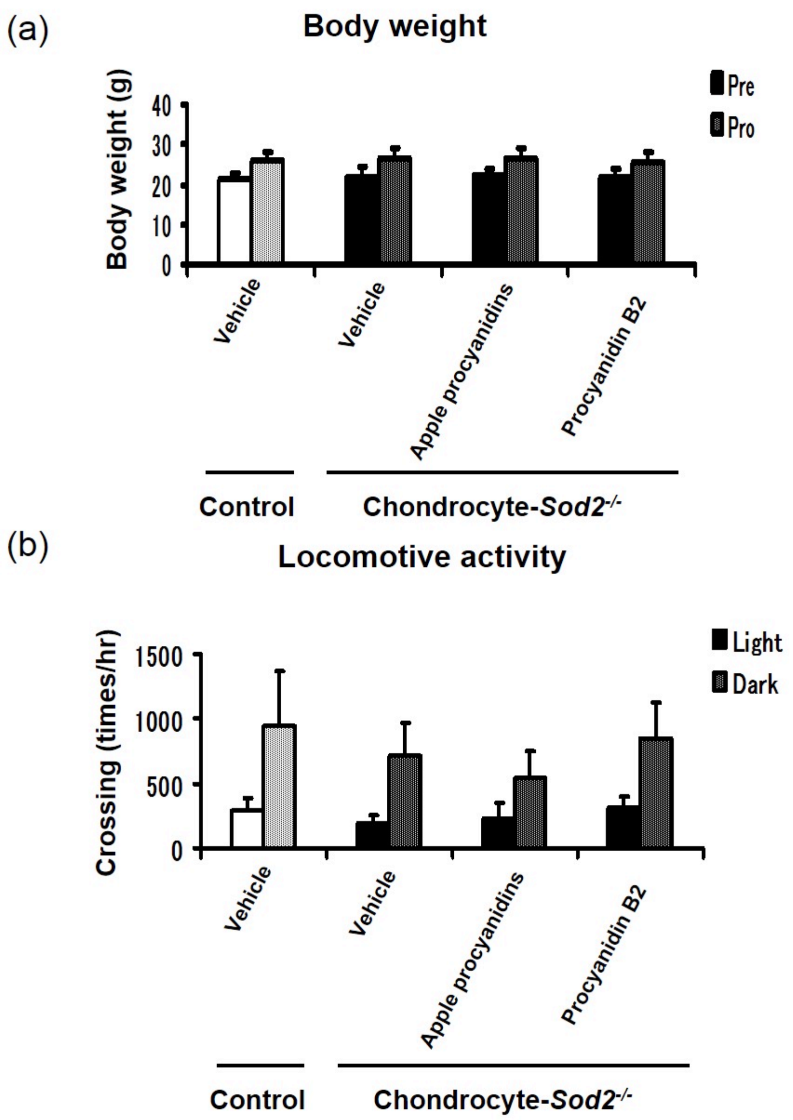

Supplementary Figure S4. Apple procyanidins or procyanidin B2 do not influence the mechanical stress of chondrocyte-*Sod2*$^{-/-}$ mice under an unstable condition. Mice were treated with DMM surgery at 8 weeks of age and administered 500 mg/kg apple procyanidins or 100 mg/kg procyanidin B2 for 8 weeks. (a) Body weight. Values are the mean ± standard deviation (n = 11-12, versus control, Student's *t*-test), (b) Locomotive activity. Values are the mean ± standard deviation (n = 8, versus control, Student's *t*-test).

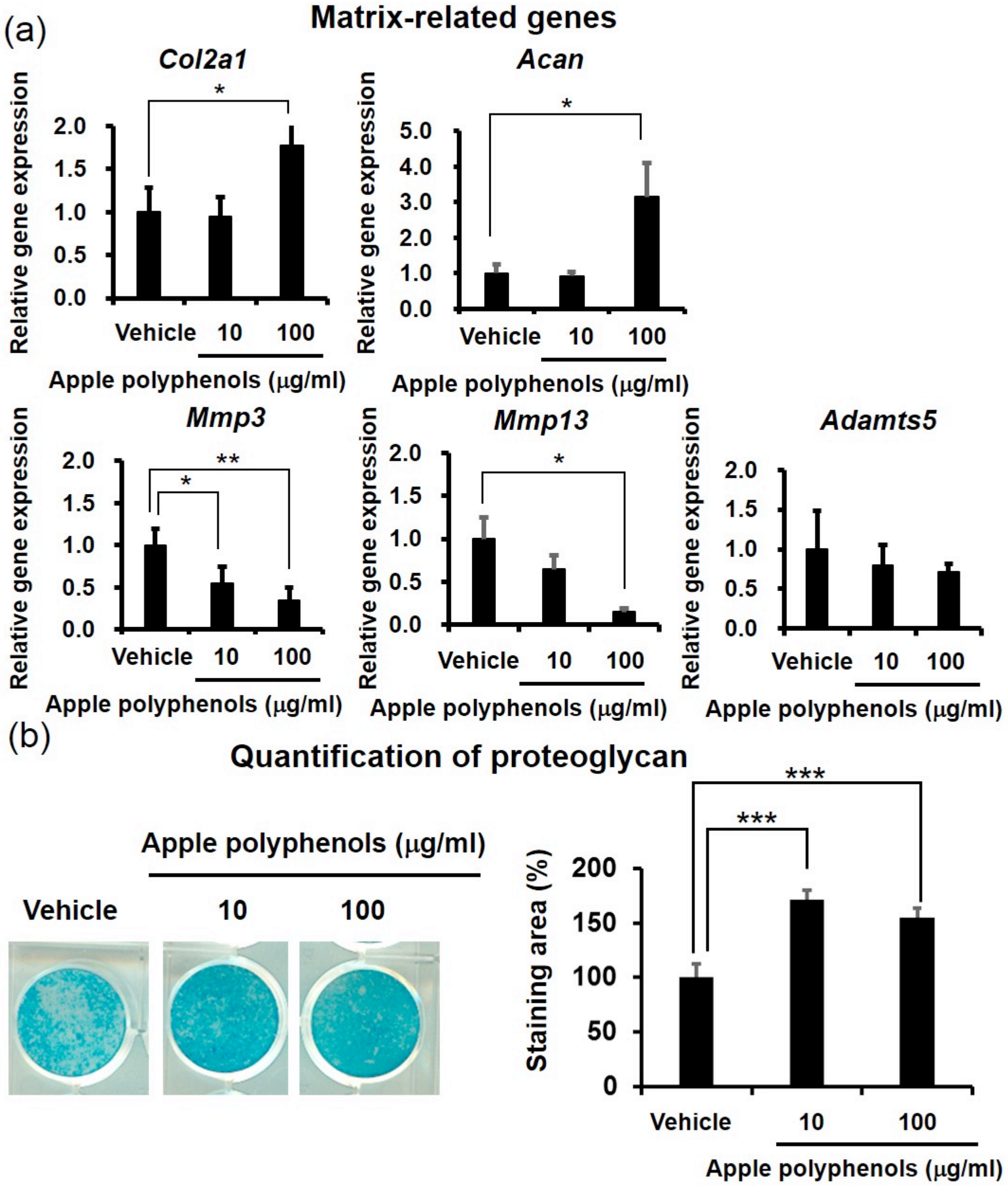

Supplementary Figure S5. Apple polyphenols enhance proteoglycan synthesis in *Sod2*-deficient chondrocytes. Apple polyphenols were added to *Sod2*$^{-/-}$ chondrocytes for 24 h for the assessment of their effects on [Please check this.] the expression of matrix-related genes and for 14 days for the assessment of their effects on [Please check this.] proteoglycan levels. (a) The expression profiles of matrix-related genes (n = 3), (b) proteoglycan levels (n = 4-6). Values are the mean ± standard deviation (*P < 0.05, **P < 0.01, ***P < 0.001 versus control, Student's t-test).

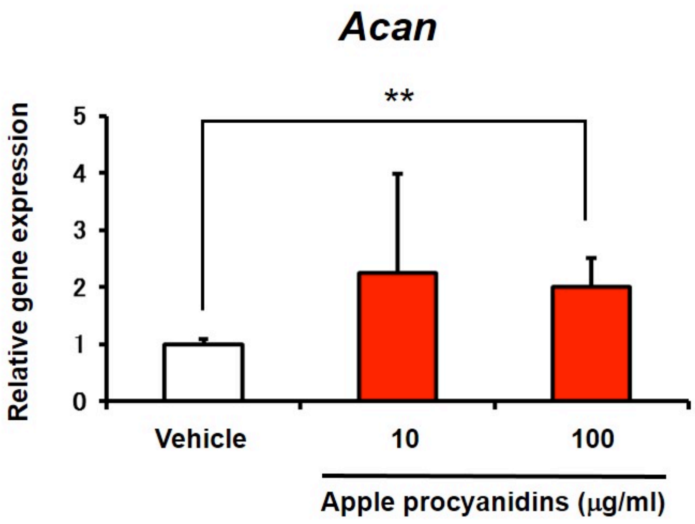

Supplementary Figure S6. The procyanidins fraction of apple polyphenols also shows enhanced proteoglycan synthesis in primary chondrocytes. Apple procyanidins were added to primary chondrocytes for 24 h. Acan Expression. Values are the mean ± standard deviation (n = 4, $^{**}P < 0.01$ versus control, Student's *t*-test).

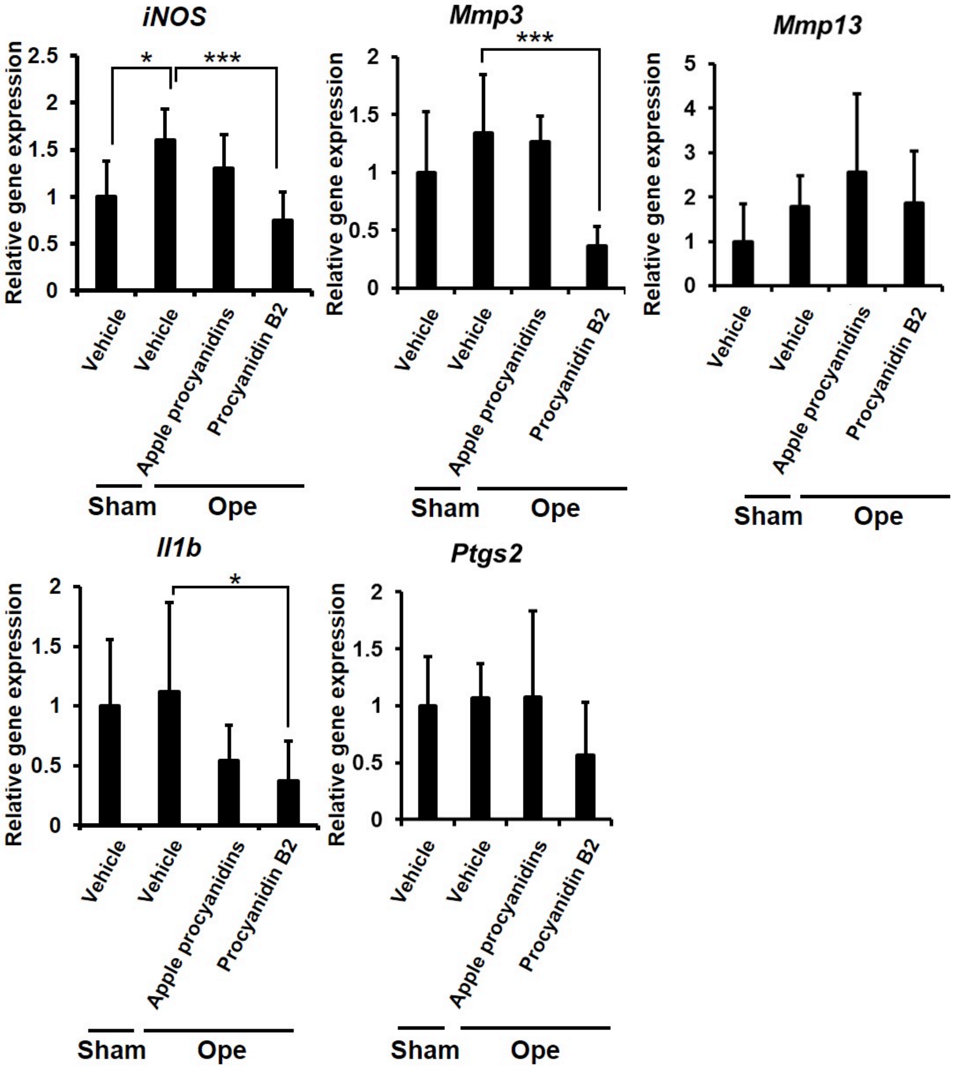

Supplementary Figure S7. Procyanidin B2 partially downregulated the expression of OA-related genes in the infrapatellar fat pad of chondrocyte-*Sod2*$^{-/-}$ mice. The left knee joints of mice were treated with DMM surgery at 8 weeks of age and administered 500 mg/kg apple procyanidins or 100 mg/kg procyanidin B2 for 2 weeks. The right knee joints of mice were treated with *sham* operations. Expression profiles of OA-related genes in synovial tissues of knee joints. Values are the mean ± standard deviation (n= 5-7, *$P < 0.05$, **$P < 0.01$, ***$P < 0.001$ versus control, Student's *t*-test).
